# Jolkinolide B Inhibits Gastric Cancer Growth by Targeting the PANoptosis Molecular Switch Caspase-8

**DOI:** 10.7150/jca.101218

**Published:** 2024-09-30

**Authors:** Chenhui Ma, Lei Gao, Kewei Song, Baohong Gu, Bofang Wang, Weigao Pu, Hao Chen

**Affiliations:** 1 The Second Clinical Medical College, Lanzhou University, Lanzhou, China.; 2 Gansu Provincial Key Laboratory of Environmental Oncology, Lanzhou, Gansu, China.; 3 Department of Tumor Surgery, Lanzhou University Second Hospital, Lanzhou, China.

**Keywords:** natural products, jolkinolide b, gastric cancer, PANoptosis, CDX

## Abstract

**Background:** To elucidate the mechanisms by which Jolkinolide B (JB), derived from Euphorbia fischeriana, suppresses gastric cancer (GC) development, given its known potent antitumor effects and the lack of detailed understanding of its impact and molecular processes in GC.

**Methods:** The study utilized both cellular and animal models to investigate the effects of JB on GC. The GC cell lines AGS and MKN45 were used to assess JB's impact on cell growth, proliferation, migration, and invasion. Molecular techniques, including molecular docking and dynamics simulations, were employed to explore the binding interactions between JB and caspase-8. The inhibitor Z-IETD-FMK was used to examine the role of caspase-8 in JB-mediated PANoptosis. Xenograft tumor transplantation experiments were conducted to evaluate JB's effect on tumor growth and biotoxicity *in vivo*.

**Results:** JB markedly inhibited the growth, proliferation, migration, and invasion of the AGS and MKN45 GC cell lines. It induced PANoptosis in GC cells by activating caspase-8, leading to increased expression of cleaved caspase-3/7 (apoptosis), GSDMD-N (pyroptosis), and p-RIPK1 and p-MLKL (necroptosis). Molecular docking and dynamics simulations revealed that JB binds effectively to caspase-8 with a binding free energy (ΔTotal) of -34.41 kcal/mol, suggesting specific binding-induced caspase-8 activation. The inhibition of caspase-8 by Z-IETD-FMK prevented JB-mediated PANoptosis. Additionally, JB significantly reduced tumor growth in xenograft models without causing biotoxicity.

**Conclusion:** JB is a promising bioactive agent that inhibits gastric cancer growth through the activation of the PANoptosis pathway. This study highlights JB's potential as an effective therapeutic option for GC, underlining the importance of its binding interaction with caspase-8 and subsequent activation of apoptotic, pyroptotic, and necroptotic pathways.

## Introduction

Gastric cancer (GC) is one of the most common malignancies worldwide and ranks as the fourth leading cause of cancer-related deaths. This high mortality rate highlights the aggressive nature of GC and the challenges associated with its late diagnosis and treatment. The survival outcomes of patients with GC are strongly influenced by the stage of the cancer at the time of diagnosis. Patients who are diagnosed with early-stage GC and who receive prompt treatment can achieve a five-year survival rate exceeding 90% 1, 2. In contrast, patients with advanced-stage disease exhibit significantly lower survival rates, generally ranging from 20% to 30% 3. Comprehensive chemotherapy-based treatment is the predominant clinical treatment modality, yet the toxicity of chemotherapeutic drugs and their propensity to induce resistance often leads to patients discontinuing treatment due to intolerance, resulting in poor therapeutic outcomes 4. Natural products, integral to traditional medical practices, are broadly acknowledged for their therapeutic effectiveness, low toxicity, and distinctive mechanisms of action 5. In the field of pharmaceutical development, exploring natural products to identify low-toxicity active compounds remains an active area of research. Consequently, the development of new medications and the identification of new treatment strategies are critically important for increasing the efficacy of GC treatment.

There is growing evidence indicating that traditional medicine preparations and their individual components effectively control the progression of GC and extend patient survival, with components derived from Euphorbia fischeriana being representative medications 6. Euphorbia fischeriana belongs to the Euphorbiaceae family and is an herbaceous plant. Its roots are widely used as medicinal materials for treating edema, ascites, cancer, and other diseases, and jolkinolide B (JB) is one of its main components 7. Initial studies have revealed that the MAPK/ERK signaling pathway is blocked by JB, which in turn inhibits breast cancer cell migration 8.

Additionally, the proliferation and spread of lung cancer can be curbed by JB through the reduction of HK2 expression via the Akt/mTOR pathway, thereby suppressing glycolysis 9. Moreover, JB induced apoptosis in mouse melanoma B16F10 cells and inhibited tumor growth by altering glycolysis 10. Additionally, JB induced cell apoptosis by activating ROS-mediated ER stress and the ERK pathway, and it triggered two different cell death modes by inhibiting TrxR1 and depleting GSH to induce excessive ROS generation 11. The findings from these studies suggest that JB has potential therapeutic benefits in various malignant tumors, highlighting its diverse mechanisms of action and induction of multiple forms of cell death. Nonetheless, the specific therapeutic effects of JB on GC and its mechanisms still warrant additional exploration.

In our research, we conducted a thorough investigation of the antitumor effects of JB on GC and its associated mechanisms utilizing both *in vitro* and *in vivo* methodologies. We clarified that JB promotes PANoptosis in GC cells by activating a caspase-8-dependent signaling pathway. These findings offer a scientific basis for the potential clinical application of JB as a treatment for GC.

## Materials and methods

### Herbal medicine

JB was acquired from Shanghai yuanye Bio-Technology Co., Ltd., in June 2022 (37905-08-1; HPLC≥98%). JB samples were stored at the Gansu Provincial Key Laboratory of Environmental Oncology.

### Cell culture and reagents

The Gansu Provincial Key Laboratory of Environmental Oncology provided the human GC cell lines AGS and MKN45. These cells were cultivated in RPMI-1640 medium (HyClone, USA) supplemented with 10% fetal bovine serum (PAN Biotech UK Ltd.) and 1% penicillin‒streptomycin. Incubation was performed under sterile conditions, with the environment kept at a constant temperature of 37°C and a 5% CO_2_ atmosphere. Furthermore, the caspase-8 inhibitor Z-IETD-FMK was obtained from MedChemExpress (MCE, HY-101297).

### CCK-8 assays

Following digestion, the cell suspension was diluted, and approximately 7,000 cells per well in 100 µL of media were seeded into a culture plate. Three replicate wells were established for each experimental group. After seeding, the cells were placed in a CO_2_ incubator for 24 hours to promote adhesion. Following this, JB was administered to each well at varying concentrations as required by the experiment, and the cells were incubated again 10 µL of CCK-8 solution was added to each well, and the cells were incubated for 1-4 hours. Following this incubation, absorbance was measured at 450 nm using a microplate reader, with readings taken every hour.

### Migration assays

A wound healing assay was used to analyze the migration capacity of AGS and MKN45 cells. When the cells reached the optimal density, a standardized scratch was applied to the monolayer cultures using a 1 ml pipette tip. The cells were then incubated with JB (15 μM for AGS and 30 μM for MKN45) for 24 hours. To observe and quantitatively assess wound closure, six random fields were chosen and examined under a microscope at various time intervals.

### Invasion assays

Matrigel was prepared at a concentration of 1 mg/mL and 50 μL was added to each well to coat the polycarbonate membranes, followed by a gelation incubation period. In the experimental design, each well in the lower chamber was supplemented with 600 μL of culture medium containing 10% fetal bovine serum, and 100,000 AGS and MKN45 cells were seeded in the upper chamber. The upper chamber was then incubated for 24 hours with various concentrations of JB (15 μM for AGS and 30 μM for MKN45). Following incubation, the chambers were washed with PBS. To assess the invasive capabilities of the cells, a meticulous process was followed. Cells that successfully migrated through the membrane were fixed, stained, and then examined under an inverted microscope. Cells that remained stationary on the upper surface of the membrane were carefully removed using a cotton swab. In contrast, this procedure enabled the precise counting and evaluation of only the cells that demonstrated the ability to migrate through the membrane.

### Colony formation assays

AGS and MKN45 cells were seeded into six-well plates, each well containing approximately 800 cells. The cells were maintained at 37°C in an atmosphere of 5% CO_2_ for 24 hours to facilitate optimal adhesion after being submerged in 2 mL of RPMI-1640 medium containing 10% FBS. The cells were treated with various concentrations of JB (AGS: 15 μM; MKN45: 30 μM) after incubation, and the media was changed every three days during a cultivation period of 10-12 days. Staining with 0.5% crystal violet was then performed on the cells at ambient temperature. Colonies with more than 50 cells per well were quantified and evaluated with ImageJ software.

### Live/dead costaining

The cells were initially incubated with JB (15 μM for AGS and 30 μM for MKN45) for 24 hours. Following incubation, a calcein-AM/PI dual staining kit (NO. 40747ES76, Yeasen, Shanghai, China) was utilized to distinguish between live and dead cells identified using a fluorescence microscope. Live cells emitted green fluorescence, while dead cells exhibited red fluorescence.

### Cell apoptosis assays

To investigate cell death before and after treatment with JB, cells were harvested from both the untreated control and JB-treated experimental groups after 24 hours of exposure using EDTA-free trypsin followed by several PBS washes. Following initial preparations, the cells were analyzed with an Annexin V-FITC/PI double-staining apoptosis detection kit to evaluate cell death. Preparation began by suspending the harvested cells in 100 μL of 1× binding buffer within flow cytometry tubes. To this suspension, 5 μL of Annexin V-FITC and 10 μL of PI were added. The cell mixture was incubated at room temperature in the dark for 10-15 minutes. Subsequently, 400 μL of 1× binding buffer was added. Finally, the samples were examined via flow cytometry, which took place within an hour of completing the preparation.

### Transmission electron microscopy (TEM)

GC cells were initially cultured in 6-well plates. After cultivation, the cells were treated with a specific concentration of JB (15 μM for AGS and 30 μM for MKN45) for 24 hours. Following treatment, the cells were collected and subsequently fixed in a 2.5% glutaraldehyde solution. The fixing process was followed by a staining phase using osmium tetroxide. After staining, the cells were dehydrated through a graded series of acetone solutions before being embedded in epoxy resin. The preparation, examination, and imaging of the TEM samples were conducted at the Biomedical Imaging Research Platform of the Cuiying Biomedical Research Center at Lanzhou University.

### Western blotting

The levels of proteins involved in the PANoptosis signaling pathway, including cleaved caspase-8, cleaved caspase-3/7, GSDMD-N, p-RIPK1, and p-MLKL, were determined. We conducted this analysis using Western blotting to assess the levels of these proteins. First, total protein from AGS and MKN45 cells was extracted using lysis buffer containing PMSF and stored at low temperature. After the cells were treated with PBS, they were added to lysis buffer and sonicated for disruption, followed by low-temperature centrifugation to separate the supernatant.

Protein samples were denatured using SDS loading buffer and subsequently stored at -80°C. Protein concentrations were determined via the BCA assay. Based on the protein mass, electrophoresis of the samples was performed, followed by SDS‒PAGE and subsequent wet transfer of the proteins onto a PVDF membrane. After membrane blocking, sequential incubation with primary and secondary antibodies was conducted, followed by visualization using an enhanced chemiluminescence (ECL) substrate, and the results were recorded using an imaging system. The following antibodies were used: β-actin (Proteintech, 81115-1-RR. 1:5000). Cleaved caspase 3 (Asp175) (CST, 5A1E. 1:1000); and cleaved caspase-7 (Asp198) (CST, D6H1. 1:1000); cleaved caspase 8 (Asp374) (CST, 18C8, 9496. 1:1000); cleaved gasdermin E (Asp270) (CST, E8G4U, 55879. P-RIPK1 (S166) (Proteintech, 28252-1-AP. 1:2000); p-MLKL (phospho S345) (Abcam, ab196436. 1:1000).

### RNA sequencing

Total RNA was extracted from MKN45 cells before and after JB (30 μM) intervention for 24 hours, and RNA sequencing analysis was performed. High-throughput mRNA sequencing was conducted at Majorbio Biotechnology Co., Ltd.

### *In vivo* safety assessment of JB

Healthy NOD-SCID mice were subjected to a treatment regimen involving JB. The compound was delivered through intraperitoneal injection at a concentration of 120 mg/kg. JB was administered once every two days, totaling four consecutive administrations. After the final injection, the mice were euthanized for further analysis. Whole blood was obtained using the eye bleeding method, and the samples were centrifuged at 800 × g for 10 minutes to separate the serum, which was then sent to Wuhan Saiweier Biotechnology Co., Ltd., for biochemical analysis.

### Construction of the xenograft tumor model

To develop a GC xenograft model, we used female NOD-SCID mice. The mice chosen for this study were aged between 5 and 6 weeks. When the confluence of MKN45 cells reached 80-90%, enzymatic digestion was performed using trypsin, followed by washing in PBS and subsequent adjustment of the cellular concentration to 5×10^7 cells/mL. Under sterile circumstances, the fur of the mice was meticulously shaved and the skin was sterilized using iodine. Subsequently, a 0.1 mL volume of cell suspension was administered subcutaneously into the right hind limb. Mouse and tumor growth monitoring was conducted, and mice with evenly growing tumors were chosen for subsequent experiments. Mice were subjected to a treatment regimen involving JB. The compound was delivered through intraperitoneal injection at a concentration of 40 mg/kg. JB was administered once every two days, totaling four consecutive administrations. After the final injection, the mice were euthanized for further analysis.

### H&E staining

Tissues were fixed in a 4% paraformaldehyde solution for more than 24 hours to preserve cellular morphology and structure. Tissue sections were embedded in paraffin and sectioned at a thickness of 8 µm. Next, the sections were subjected to H&E staining. For the purpose of evaluation, an optical microscope from Olympus, Japan, was used to analyze the stained sections.

### Immunohistochemistry (IHC)

Initially, the samples were deparaffinized and subjected to antigen retrieval. Subsequently, endogenous peroxidase activity was inhibited using a 3% solution of hydrogen peroxide applied at 25°C for 10 minutes. To minimize nonspecific binding, the specimens were subjected to treatment with serum for 30 minutes. The specimens were then incubated overnight at 4°C with primary antibodies, followed by a 30-minute incubation at 25°C with secondary antibodies. After DAB staining, color development was terminated by washing, followed by counterstaining, dehydration, clearing, mounting, and analysis.

### Molecular dynamics and stability assessment

We used Gromacs 2022.3 to perform molecular dynamics simulations on the protein‒ligand complexes obtained through molecular docking. AmberTools22 was used to add the general AMBER force field (GAFF), and Gaussian 16W was employed for hydrogenation and restrained electrostatic potential (RESP) calculations of small molecules to optimize geometric configurations and charge distribution. The simulation conditions were set to 300 K and 1 Bar, using the Amber99sb-ILDN force field and Tip3p water model, and Na+ ions were added to neutralize the system. We initially used the steepest descent method for energy minimization and then conducted NVT and NPT equilibrations, each for 100,000 steps, lasting 100 ps. Finally, we conducted free molecular dynamics simulations for a total of 50,000,000 steps with a step size of 2 fs, totaling 100 nanoseconds (ns). Analyses included root mean square deviation (RMSD), RMSF, radius of gyration (Rg), solvent-accessible protein surface (SASA), and analysis of the hydrogen bonds of the complex, which provided detailed insights into the structural stability, flexibility, and solvent accessibility of the complex. We further assessed the binding energy and thermodynamic stability of the small molecule with the protein receptor using the Gibbs free energy landscape and molecular mechanics/generalized Born surface area (MM/GBSA) methods.

### Caspase-8 inhibitor assay

To investigate the role of Caspase-8 activation in the PANoptosis pathway, AGS and MKN45 cells were first pre-treated with 40 µM of the Caspase-8 inhibitor Z-VAD-FMK for 1 hour. Following this pre-incubation, the cells were exposed to varying concentrations of JB (15 μM for AGS cells and 30 μM for MKN45 cells) for 24 hours. Subsequent assessments of cell viability and apoptosis were performed. Additionally, key proteins involved in PANoptosis were analyzed via Western blotting.

### Statistical analysis

The experimental outcomes are presented as the mean ±the standard deviation (mean ± SD). Each test was replicated three times (n=3). The analysis of the data utilized IBM SPSS software version 23.0. Statistical thresholds were set at *P<0.05 for significance, **P<0.01 for high significance, and ***P<0.001 for very high significance, with nonsignificant results marked as NS.

## Results

### Effects of JB on the viability, proliferation, migration, and invasion of AGS and MKN45 GC cells

**Figure 1:**
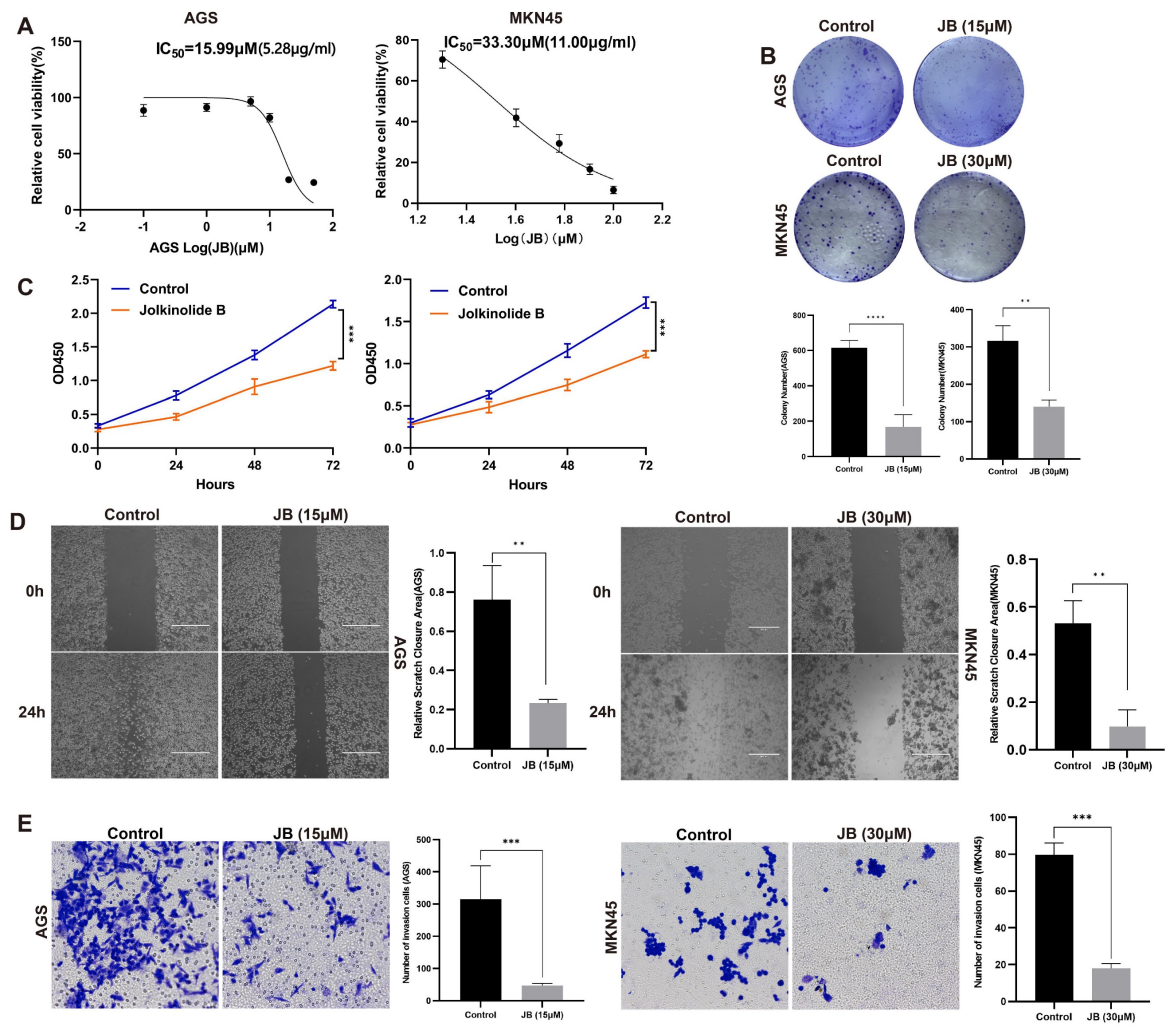
** JB effectively inhibits the viability, proliferation, migration and invasion of GC cells.** (A) The IC50 of JB was calculated using dose-response curves of GC cell lines. The value for AGS is 15.99 μM and for MKN45 is 33.3 μM. (B) A colony formation assay was used to assess the proliferative capacity of AGS and MKN45 cells after treatment with JB at oncentrations of 15 µM and 30 µM for 24 hours. (C) After treating GC cells with or without JB, cell viability at 72 hours was assessed using the CCK-8 assay. (D) The migratory capacity of GC cells was assessed using a wound healing assay after treating the cells with or without JB for 24 hours. (E) Transwell assays were used to evaluate the invasive behavior of AGS and MKN45 cells after 24 hours of treatment with or without JB.

To assess the influence of JB on GC cell lines, its effects on cellular viability were evaluated by plotting a logarithmic dose-response curve across various concentrations (Figure 1A). The analysis revealed an IC50 value of 15.99 μM (5.28 μg/ml) in AGS cells and 33.30 μM (11.00 μg/ml) in MKN45 cells. The sensitivity of AGS cells to JB was greater than that of MKN45 cells. In the colony formation assay (Figure 1B), both AGS and MKN45 GC cell lines showed significantly reduced colony formation ability after JB treatment. The findings demonstrated that JB significantly impedes the clonal proliferation of GC cells *in vitro*, further corroborating its anticancer efficacy. AGS and MKN45 cells were treated with JB at specific concentrations for 24-72 hours. JB treatment notably reduced the proliferation of AGS and MKN45 GC cells. As shown in Figure 1C, the AGS and MKN45 groups exhibited a significant reduction in cell numbers at various time points compared to the control group. These findings suggest that JB effectively decreases the proliferative activity of GC cells, with the effect strengthening over time (P<0.001). In the scratch and invasion assays conducted on AGS and MKN45 GC cell lines (Figure 1D-E), JB significantly inhibited cell migration and invasion, providing a basis for further research into the role of JB in GC therapy.

### JB promotes apoptosis and induces morphological changes in GC cells

**Figure 2:**
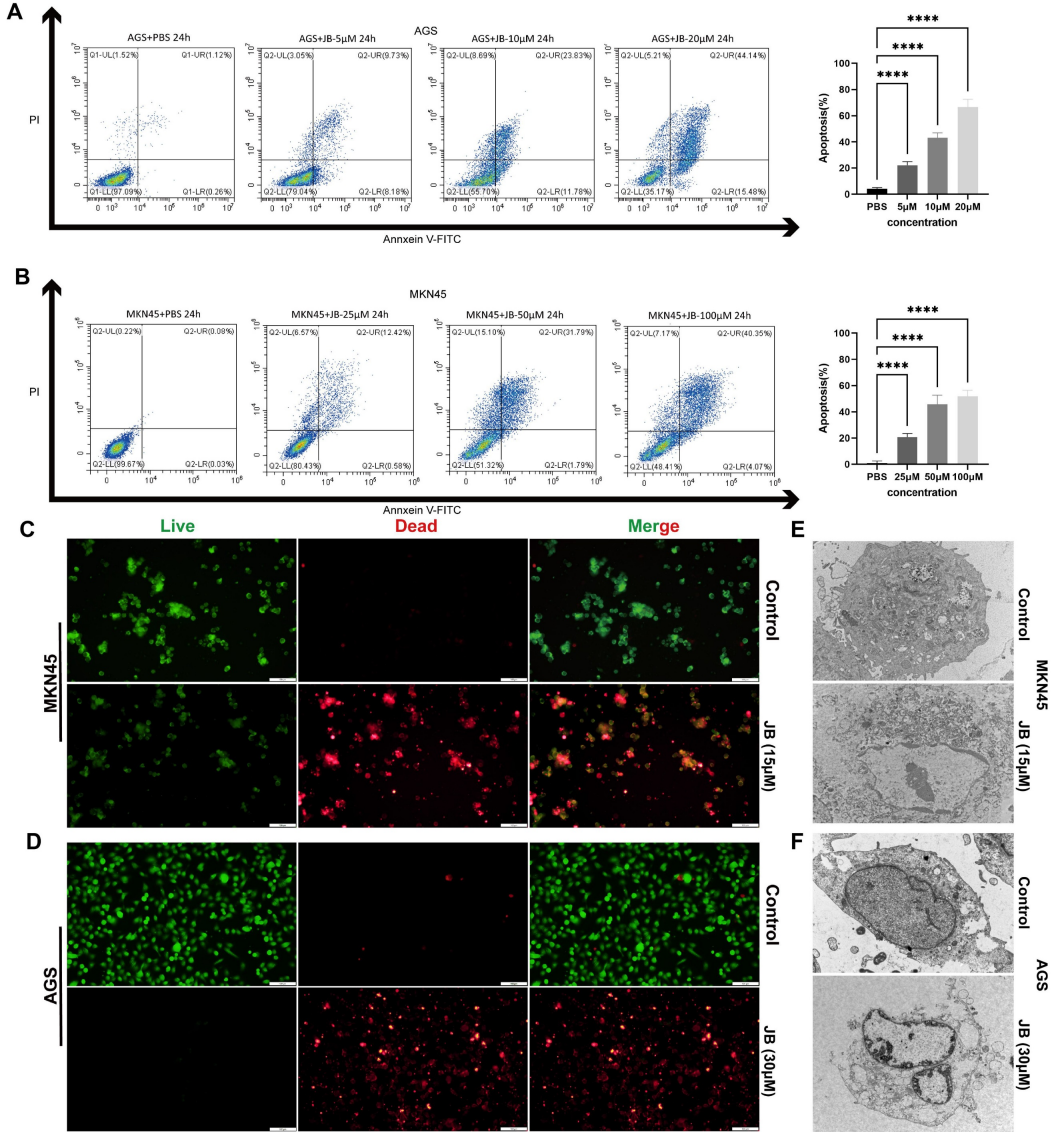
** JB promotes apoptosis and induces morphological changes in GC cells.** (A-B) The apoptotic rates of AGS and MKN45 cells treated with various concentrations of JB were determined using Annexin V-FITC/PI double staining. (C-D) The viability and death of AGS and MKN45 cells before and after 24-hour treatment with JB (15 µM for AGS cells and 30 µM for MKN45 cells) were assessed using calcein-AM/PI live/dead cell staining. (E-F) Morphological changes in AGS and MKN45 cells before and after 24 hours of JB treatment were observed using TEM.

Following the application of Annexin V-FITC and PI staining, flow cytometry analysis illustrated that JB significantly increased apoptosis in AGS and MKN45 GC cell lines, with effects that intensified with greater concentrations. Significant apoptotic effects were observed at concentrations of 20 μM in AGS cells and 100 μM in MKN45 cells. The percentage of apoptotic cells was markedly higher in the groups treated with elevated concentrations of JB compared to the control group treated with PBS, as shown in Figures 2A-B (P<0.0001). For direct observation of cell viability, live/dead cell staining techniques were employed via the use of calcein AM to label viable cells in green and PI to label nonviable cells in red. The findings depicted in Figures 1C-D illustrate significant alterations resulting from JB treatment: a noticeable decrease in the number of green fluorescent live cells, accompanied by an increase in the number of red fluorescent dead cells, was observed. These data further support the cytotoxic properties of JB. Additionally, ultrastructural changes in the MKN45 and AGS GC cell lines induced by JB were demonstrated via TEM. In the control group, MKN45 and AGS cells displayed the following typical ultrastructural characteristics of GC cells: regular cell morphology, intact and continuous cell membranes, large nuclei with partially lobulated structures, and clearly visible nucleoli. In contrast, both cell lines treated with JB showed significant ultrastructural changes. Specifically, under the effect of JB, MKN45 (Figure 2E) and AGS cells (Figure 2F) exhibited organelle swelling, cell membrane rupture, and extensive vacuolation. Moreover, organelles were degraded to the point of being unrecognizable, nuclei were condensed or fragmented, and cell membranes exhibited ruptures or pore formation, leading to the release of intracellular contents. These findings imply that JB could play a critical role in promoting apoptosis and/or necrosis in GC cell lines.

### Effects of JB intervention on mRNA and protein expression were evaluated by RNA sequencing and western blotting

**Figure 3:**
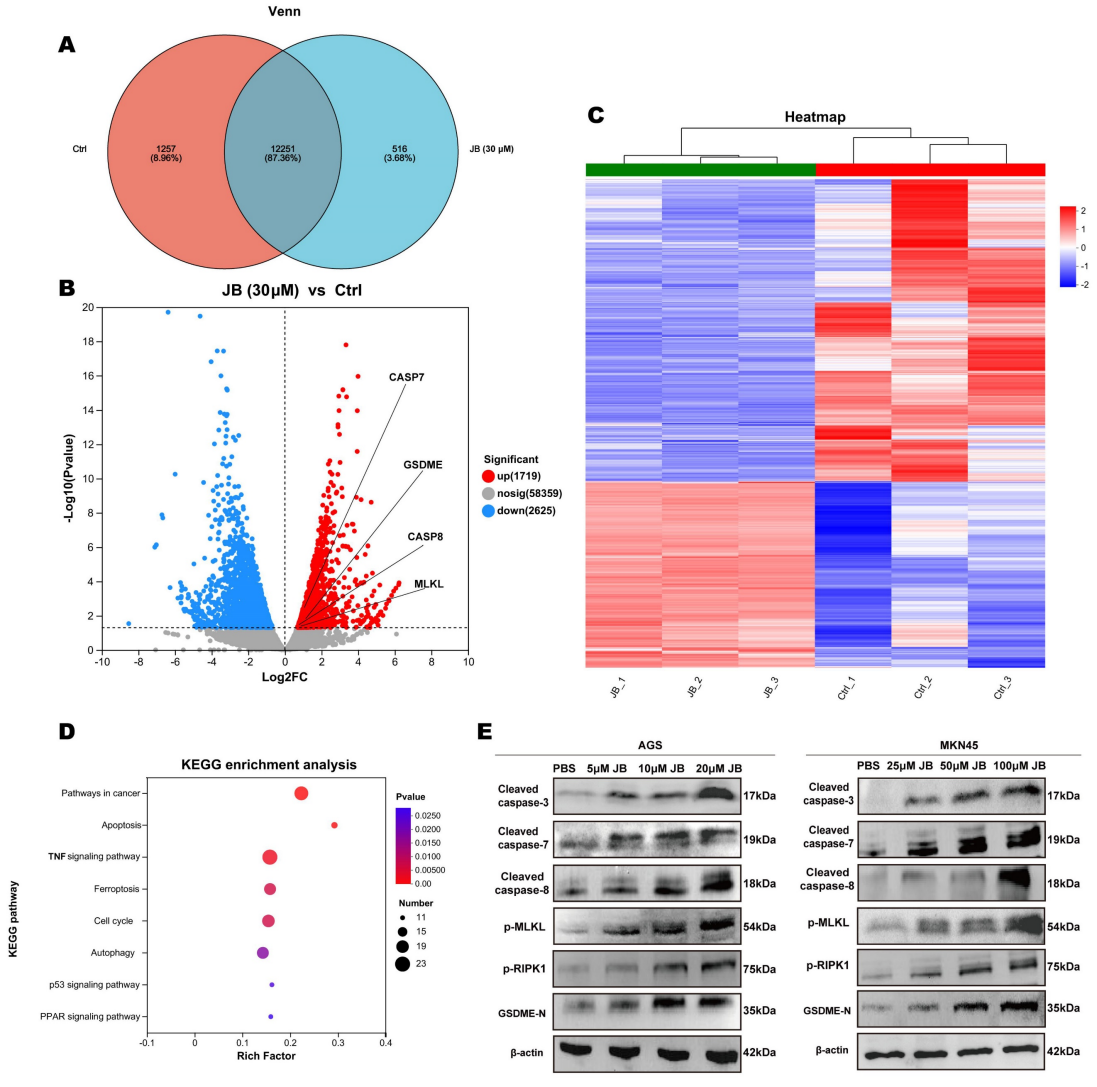
** RNA sequencing and Western blotting were performed before and after JB intervention.** (A) Venn diagram displaying the genes expressed in MKN45 cells before and after 24 hour JB treatment at 30 µM. (B) A volcano plot illustrates the differentially expressed genes between control and JB-treated MKN45 cells (30 µM, 24 hours). (C) A heatmap illustrates the differentially expressed genes between JB-treated and control MKN45 cells. (D) KEGG enrichment analysis of differentially expressed genes. (E) Changes in key proteins in GC cells treated with varying concentrations of JB were analyzed using Western blotting.

Figure 3A displays the results of transcriptome sequencing performed on untreated (control group) and JB-treated MKN45 GC cells. By comparing gene expression differences between the two groups, this study aimed to elucidate the cellular response mechanisms induced by JB. The results revealed that in the control group, 1257 genes were transcribed, while in JB-treated cells, 516 genes were transcribed. As illustrated in Figure 3B, JB treatment resulted in the upregulation of 1719 genes and the downregulation of 2625 genes compared to the control. Specifically, genes related to the apoptotic process, such as caspase-8, caspase-7, GSDME, and MLKL, were significantly upregulated (P<0.05). Figure 3C presents a heatmap of all differentially expressed genes, offering a visual representation of the overall shifts in gene expression patterns. Additionally, KEGG analysis between the two groups (Figure 3D) revealed that pathways related to cell death, including pathways related to cancer, apoptosis, ferroptosis, autophagy, and the p53 signaling pathway were enriched in the differentially expressed genes (P<0.05), thus providing insights into the mechanisms by which JB induces cell death. To further demonstrate the diversity of cell death processes post-JB intervention, we focused on PANoptosis and validated its key proteins using Western blotting, as shown in Figure 3E. After treatment of AGS and MKN45 cells with a concentration gradient of JB, the expression of cleaved caspase-8, cleaved caspase-3/7, p-RIPK1, p-MLKL and GSDME-N increased in a concentration-dependent manner.

### Validation of the safety and efficacy of JB through *in vivo* experiments

**Figure 4:**
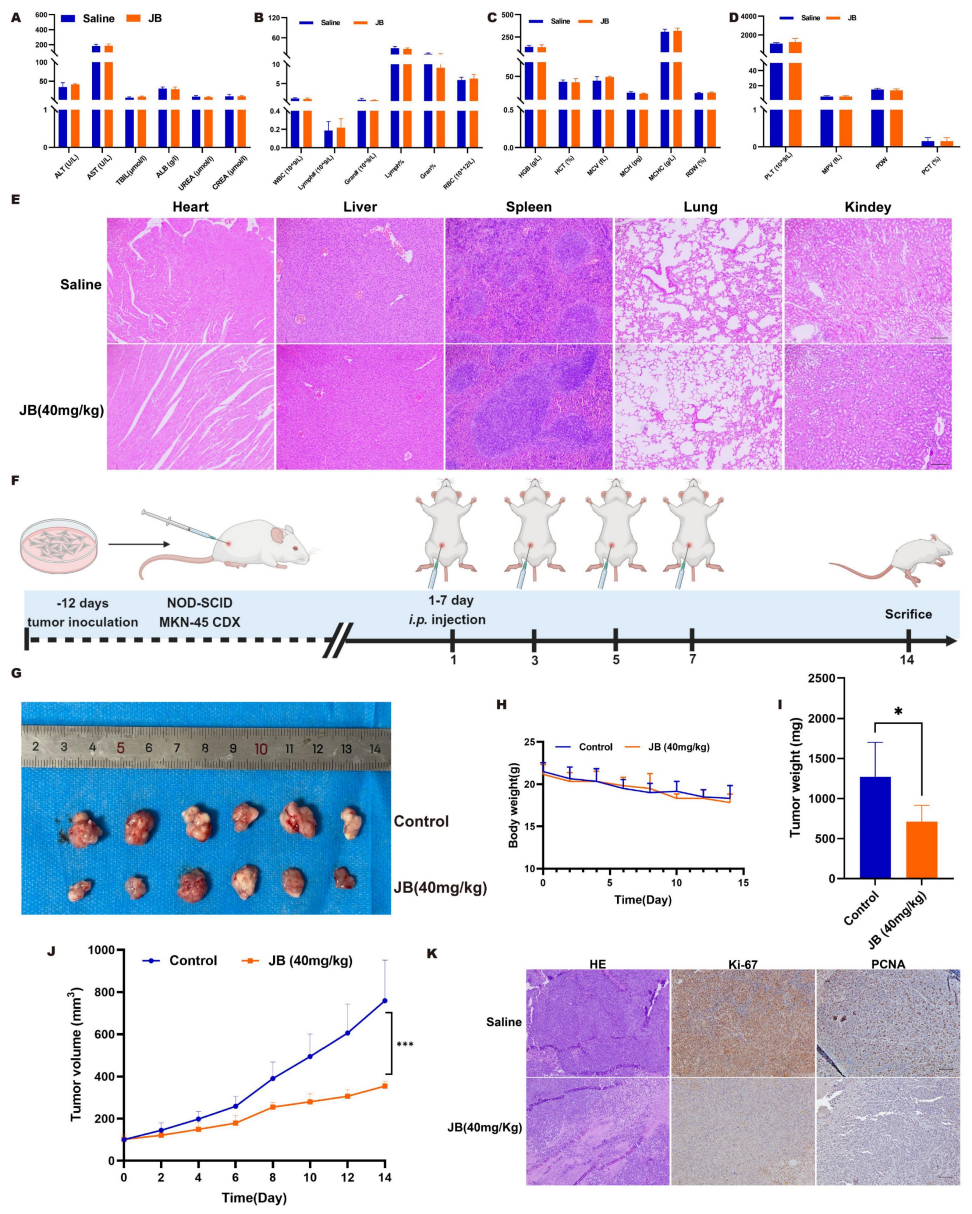
**Safety and efficacy of JB were validated through *in vivo* experiments.** (A-D) After the intraperitoneal injection of JB into normal NOD-SCID mice, blood samples were collected for complete blood cell analysis, and liver function and renal function tests. (E) Important organs from mice were harvested for histological examination. H&E staining was used to observe tissue damage. (F) Preparation of GC xenograft mice and the intervention protocol with JB. (G) Photographs of tumor tissues dissected from euthanized CDX model mice. (H-I) Body weight changes in the JB-treated and control mice. (J) Changes in the volume of subcutaneous tumors in JB-treated and control mice. (K) H&E, Ki67, and PCNA staining of *ex vivo* tumor sections from JB-treated and control group mice.

Prior to *in vivo* antitumor treatments, the toxicological impact of JB on mice was evaluated, starting with its effects on blood biochemical markers of liver and kidney function. The parameters measured included the levels of ALT, AST, TBil, Alb, blood urea nitrogen, and creatinine. Postadministration of JB, no deviations in liver or kidney functions were observed in the mice. Comprehensive hematological profiles encompassing WBC count, Lymph count, Gran count, RBC count, HGB levels, HCT levels, MCV, MCH, MCHC, RDW, PLT count, MPV, PDW, and PCT levels revealed that JB did not markedly influence hematological parameters related to red blood cells or platelets in mice. Following the experiments, major organs were extracted from the mice and subjected to H&E staining for histological analysis. Comparative observations of the toxicity of JB on major organs revealed normal nuclear morphology and cell structure and no significant tissue damage. These findings suggest that JB is relatively safe under these experimental conditions. Figure 4F illustrates the methodology used for establishing a NOD-SCID mouse MKN-45 GC xenograft model along with the JB intervention, aiming to comprehensively assess the treatment effects. The findings indicated that the growth of the subcutaneous tumors in the JB-treated group was significantly inhibited (Figure 4G, I-J).

The weight change curve of CDX model mice (Figure 4H) showed no significant decrease during the treatment process. This indicates that the drug dosage used in this study is reasonable, and each treatment was safe for use in mice. Subcutaneous tumor tissues were collected under the above experimental conditions. H&E staining indicated that necrotic characteristics were more pronounced in the tumor tissues from the JB treatment group than in those from the saline control group. Immunohistochemical analysis of Ki-67 and PCNA showed that JB treatment had a significant inhibitory effect on tumor cell proliferation (Figure 4K).

### Molecular docking analysis

**Figure 5:**
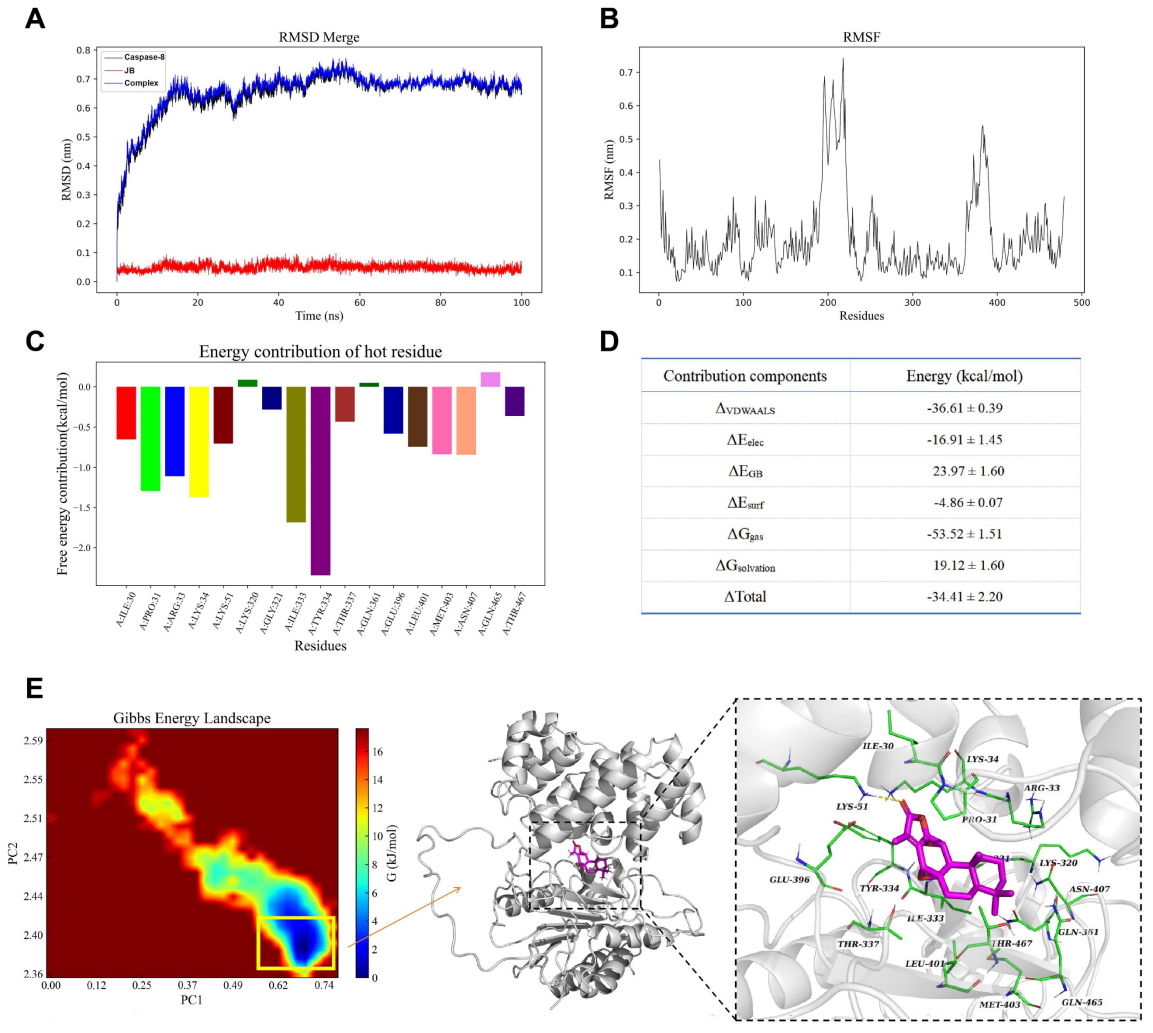
** Molecular dynamics and stability assessment between caspase-8 and JB.** (A) The RMSD of caspase-8, JB, and caspase-8-JB over time. (B) The RMSF plot of caspase-8 residues. (C) The free energy contributions of key residues during the caspase-8-JB binding process. (D) The binding free energy and its decomposition. (E) Gibbs free energy landscape and three-dimensional structure of caspase-8-JB.

Figure 5A shows the structural stability of the molecules during the simulation. The black and blue curves represent the RMSD of caspase-8 and caspase-8-JB, respectively, which rapidly increased in the first 20 ns and then stabilized in the range of 0.6-0.8 nm. The red curve represents the RMSD of JB, which remained low and stable at approximately 0.1 nm, indicating that the ligand maintained a stable conformation within the binding site. Figure 5B shows the RMSF plot of the caspase-8 residues, indicating that certain regions of the protein (such as residues approximately 150 to 250) exhibit higher RMSF values, suggesting significant conformational changes in these regions, while other regions remain more rigid and stable. Figure 5C shows the free energy contributions of key residues during the binding process of caspase-8 and JB, with the results indicating that LEU-333 and TYR-334 have significant negative values, suggesting their notable contributions to ligand binding, while residues such as LYS-320 and GLN-465 contribute relatively less.

Figure 5D shows the binding free energy and its decomposition obtained through MM/GBSA calculations, including van der Waals interactions (ΔVDWAALS), electrostatic interactions (ΔEele), polarization energy (ΔEGB), and nonpolarization energy (ΔEsurf). The total binding free energy (ΔTotal) was -34.41 kcal/mol, indicating high binding affinity between the protein and ligand, with ΔVDWAALS and the ΔG nonpolar solvation energy (ΔGsolvation) contributing the most to the binding free energy, at -36.61 and -53.52 kcal/mol, respectively. Figure 5E shows the Gibbs free energy landscape and the three-dimensional structure of caspase-8-JB at the lowest energy point, with the structure corresponding to the lowest energy point extracted and shown in the three-dimensional structure diagram on the right. Within this structure, a crucial hydrogen bond is established by the ligand with the LYS-51 site of the protein, enhancing both the stability and binding affinity of the ligand within the binding site.

### Z-IETD-FMK inhibits PANoptosis pathway activation in GC cells by suppressing caspase-8

**Figure 6:**
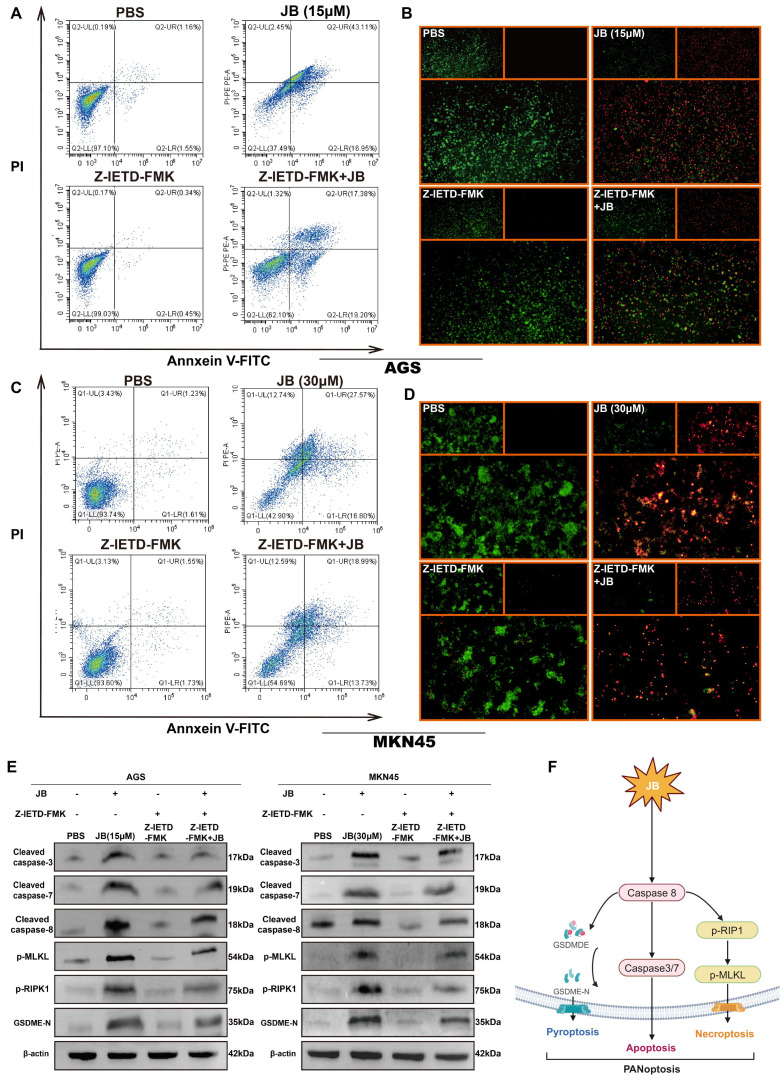
**Z-IETD-FMK inhibits the PANoptosis pathway in GC cells by inhibiting caspase-8.** (A-E) AGS and MKN45 cells pre-treated with 40 μM caspase-8 inhibitor Z-IETD-FMK for 1 hour partially reversed JB-induced cell death. Flow cytometry, live/dead cell staining, and Western blotting were used to assess changes in GC cells and key PANoptosis proteins following pre-treatment with the caspase-8 inhibitor Z-IETD-FMK, followed by JB. The results demonstrated a decrease in apoptosis rate, an increase in the proportion of viable cells, and downregulation of key proteins. (F) Schematic of the mechanism revealed in this study.

To deepen our understanding of the role of the caspase-8-dependent pathway in JB-induced PANoptosis in GC cells, experiments involving simultaneous treatment with a caspase-8 inhibitor (Z-IETD-FMK) and JB were carried out on two distinct GC cell lines. Figure 6A-D shows the effects of Z-IETD-FMK and JB on GC cell lines (AGS and MKN45). In the present study, groups treated with JB or Z-IETD-FMK alone, or a combination of both JB and Z-IETD-FMK were compared to a PBS-treated group, which was utilized as the negative control. Annexin V-FITC/PI double staining was performed to evaluate the effects of the treatments. The data from the combination treatment group indicate that Z-IETD-FMK abrogates apoptosis in AGS and MKN45 cells by inhibiting caspase-8. Similarly, the viability/cytotoxicity staining and flow cytometry results showed that Z-IETD-FMK abrogated the increased mortality of AGS and MKN45 cells induced by caspase-8. As shown in Figure 5E, Western blotting experiments demonstrated that, across the two cell lines, the combination treatment group exhibited reduced levels of cleaved caspase-3/7/8, GSDME-N, p-RIPK1, and p-MLKL compared to the JB-only treatment group. Furthermore, the model illustrated (Figure 6F) that PANoptosis induced by JB in GC cells is mediated through a caspase-8-dependent pathway.

## Discussion

Traditional medicine has complex components that, unlike modern medicines, which work through specific targets, characteristically act on multiple targets and pathways 12. *Euphorbia fischeriana* Steud., which belongs to the Euphorbiaceae family, is composed of a range of chemical constituents 13, and these constituents have multiple biological properties, including antibacterial, anti-inflammatory, antituberculosis, cardioprotective, and antitumor effects 14-17. Yan *et al.* 7 isolated seven new polycyclic diterpenes and 26 known analogs, including JB, from the roots of *Euphorbia fischeriana* and tested their therapeutic effects on five malignant tumor cell types. Using doxorubicin as a positive control, these diterpenes significantly inhibited the proliferation of human prostate cancer C4-2B cells, drug-resistant prostate cancer C4-2B/ENZR cells, and three other cell lines: colon cancer HCT-15 and RKO cells, and breast cancer MDA-MB-231 cells. Therefore, JB may become a viable candidate for cancer therapy.

The expansion and dissemination of cancer cells are critical malignant activities intimately associated with tumor relapse and metastasis, and strategically intervening in these behaviors is crucial for the effective management of GC 18. Our findings indicate that JB significantly suppresses the growth of GC cells, demonstrating its potential anti-GC effects. The proliferation and clonogenicity of GC cells are notably inhibited by JB *in vitro*, indicating its role in preventing GC cell recurrence. This investigation demonstrated that JB significantly suppressed the migration and invasion capabilities of GC cells, as evidenced by scratch tests and Transwell assays. RNA sequencing of MKN45 cells before and after JB intervention revealed enrichment of multiple KEGG pathways associated with cancer cell death. Moreover, we observed elevated expression of key genes related to apoptosis, programmed cell death, and necroptosis after JB treatment, as validated by Western blot analysis.

As identified by Professor Kanneganti in 2019, PANoptosis represents a new type of programmed cell death distinguished by its unique regulatory mechanisms and strong association with diverse diseases 19-21. PANoptosis, a form of programmed cell death characterized by its inflammatory nature, is triggered by distinct agents and regulated through the actions of the PANoptosome complex. It has features of pyroptosis, apoptosis, and necroptosis, yet it does not align exclusively with any of these pathways. 22. Research on PANoptosis in GC is scarce. Qing *et al.* 23 used machine learning to reveal the role of PANoptosis in the immunological microenvironment of GC and as a potential risk assessment tool. Significant differences in the populations of immune cells, including plasma cells, both resting and activated memory CD4+ T cells, monocytes, natural killer cells, and M2 macrophages, between the GC group and the control group were revealed by the results. Immune cells, by participating in antibody production, influencing tumor growth, impacting therapeutic outcomes, and modulating cytokines, play a crucial role in the tumor microenvironment. Their involvement is vital for increasing the efficacy of treatment approaches for GC, highlighting their significant regulatory function. Liu *et al.* 24 identified five characteristic PANoptosis genes (TFF2, KRT7, CXCR6, BATF2, and MMP12), indicating that PANoptosis is a reliable predictor of prognosis in GC patients. Pan *et al.* 25 constructed a GC cohort using a PANoptosis gene list comprising 66 genes, including 26 genes involved in pyroptosis, 32 genes involved in apoptosis, and 8 genes involved necroptosis, and achieved similar results. Lin *et al.* 26 discovered that YBX1 increases the resistance of GC cells to oxaliplatin through the inhibition of PANoptosis. This process involves the key regulatory proteins PPM1B and USP10, which are essential for modulating the phosphorylation and ubiquitination of YBX1. Consequently, these modifications impact YBX1 protein expression and functionality, influencing both PANoptosis and oxaliplatin resistance in GC cells. These insights establish a basis for devising novel therapeutic approaches aimed at mitigating chemoresistance in GC.

In this process, members of the cysteine aspartate protease family play a crucial role in controlling host cell death, innate immune responses, and internal balance. Caspase-8 plays multiple roles within the cell and is the central signaling protein in PANoptosis. Additionally, caspase-8 is known to initiate apoptosis via death receptors, including TNFR1, and to suppress necroptosis by inhibiting kinases such as RIPK3 and MLKL. Studies have demonstrated that caspase-8 can directly or indirectly stimulate the activation of proteases such as caspase-1 and caspase-3, culminating in GSDMD/E-mediated pyroptosis 27. This finding suggested that Caspase-8 acts as a universal molecular regulator governing apoptosis, necroptosis, and pyroptosis 28. In our study, by inhibiting caspase-8 activity with Z-IETD-FMK, we noted that three modes of death were simultaneously impacted. Chen *et al.* 29 discovered that lobaplatin stimulated ribosome formation in nasopharyngeal carcinoma cells, subsequently initiating caspase-3/GSDME-mediated pyroptosis. Nevertheless, this process was diminished when Z-IETD-FMK is applied.

Growing evidence suggests that caspase-8 is instrumental in the occurrence and progression of cancer. Acting as an interlinking regulatory protein, caspase-8 orchestrates several types of cell death processes 28. This study has certain limitations. Although JB treatment markedly decreased the malignant behavior of GC cells and curtailed tumor growth, these findings were confirmed only in two human GC cell lines and one subcutaneous tumor model.

## Conclusion

JB has potent anticancer effects in both cell and live animal models, significantly inhibiting the activity, proliferation, migration, and invasion of GC cells. Additionally, it activates the PANoptosis pathway involving Caspase-8 in GC cells. This research establishes a foundation for systematically exploring the effects of JB on PANoptosis in GC cells and provides theoretical and experimental groundwork for preventing and treating GC and developing JB-based medications.
